# *Cryptosporidium* infection and associated factors among diarrheic children under five years of age in Eastern Ethiopia

**DOI:** 10.1371/journal.pntd.0013386

**Published:** 2025-08-05

**Authors:** Degu Abate, Rea Tschopp, Berhanu Seyoum, Yadeta Dessie, Mahlet Osman Hasen, Gizachew Gemechu, Øystein Haarklau Johansen, David Carmena, Lucy J. Robertson, Kurt Hanevik, Alemseged Abdissa

**Affiliations:** 1 School of Medical Laboratory Sciences, College of Health and Medical Sciences, Haramaya University, Harar, Ethiopia; 2 Armauer Hansen Research Institute, Addis Ababa, Ethiopia; 3 Swiss Tropical and Public Health Institute, Kreuzstrasse Allschwil, Switzerland; 4 School of Public Health, College of Health and Medical Sciences, Haramaya University, Harar, Ethiopia; 5 Microbiology Laboratory, Southern Health and Social Care Trust, Portadown, Northern Ireland, United Kingdom; 6 Department of Clinical Science, University of Bergen, Bergen, Norway; 7 Parasitology Reference and Research Laboratory, Spanish National Centre for Microbiology, Majadahonda, Spain; 8 Centre for Biomedical Research Network in Infectious Diseases (CIBERINFEC), Health Institute Carlos III, Madrid, Spain; 9 Parasitology, Faculty of Veterinary Medicine, Norwegian University of Life Sciences, Ås, Norway; 10 National Center for Tropical Infectious Diseases, Haukeland University Hospital, Bergen, Norway; University of Washington, UNITED STATES OF AMERICA

## Abstract

**Background:**

*Cryptosporidium* infection causes diarrhea that may lead to malnutrition, growth faltering, cognitive impairment, and mortality if left untreated. Cryptosporidiosis continues to be a significant public health issue in low-income countries, including Ethiopia. However, its epidemiology among children under five years of age remains understudied. Therefore, this study aimed to determine the prevalence and factors associated with *Cryptosporidium* infection among diarrheic children under five in Eastern Ethiopia. A cross-sectional study was conducted among children under five years of age attending health centers with diarrhea in Eastern Ethiopia between November 2022 and October 2023. Fecal specimens were analyzed by auramine-phenol staining using light-emitting diode fluorescence microscopy. A structured questionnaire was used to obtain information on sociodemographic and other variables potentially associated with *Cryptosporidium* infection. Poisson regression with a robust variance model was employed to assess factors associated with *Cryptosporidium* infection using the prevalence ratio with 95% confidence intervals (CI).

**Results:**

This study included 756 diarrheic children under five years of age (mean = 20.8 months with a standard deviation of 13.4 and median of 16 months). The prevalence of *Cryptosporidium* infection was 15.2% (95% CI: 12.7–17.9). Wet season (adjusted prevalence ratio (APR) = 1.7, 95% CI: 1.2–2.4), having caregivers with no formal education (APR = 2.6, 95% CI: 1.1–6.3), presence of a diarrheic member in the household (APR = 1.9, 95% CI: 1.2–3.2), not being exclusively breastfed (APR = 1.6, 95% CI: 1.1–2.3), lack of handwashing practice after toileting (APR = 2.8, 95% CI: 1.7–4.5), and the use of toilet paper after defecation (APR = 1.6, 95% CI: 1.6–3.3) were factors significantly associated with increased risk of *Cryptosporidium* infection.

**Conclusions:**

Cryptosporidiosis was highly prevalent in diarrheic children under five years of age in Eastern Ethiopia. Exclusive breastfeeding, improving sanitation, and ensuring proper hygiene practices are essential steps in reducing the risk of *Cryptosporidium* infection.

## Author summary

*Cryptosporidium* is a significant cause of diarrheal diseases and associated malnutrition in young children in developing countries, including Ethiopia. Despite its importance as a leading cause of diarrheal illness, there is limited published data on the prevalence and factors influencing the spread of *Cryptosporidium* in this region, with most published studies focusing on populations living with HIV. This study aimed to contribute towards filling this gap by investigating the prevalence and factors associated with *Cryptosporidium* infections among diarrheic children under five years of age in Eastern Ethiopia. Among the 756 children studied, we found a 15.2% prevalence of *Cryptosporidium* infection, diagnosed using auramine-phenol staining and light-emitting diode fluorescence microscopy. We identified key factors associated with infection, including the wet season, lack of exclusive breastfeeding, inadequate handwashing after toileting, and the use of toilet paper following defecation. These findings underscore the need for targeted interventions that address these factors in order to reduce the burden of *Cryptosporidium* infections and improve child health outcomes in the region.

## Introduction

Diarrhea remains a leading cause of morbidity and mortality among children under five years of age in sub-Saharan Africa, including Ethiopia [1]. Inadequate sanitation, limited access to clean water, and insufficient healthcare infrastructure contribute to the high prevalence of diarrheal diseases [2]. Diarrhea in children under five years of age can be caused by enteric viruses, bacteria, and parasites [3,4]. Among these, the intracellular protozoan parasite *Cryptosporidium* spp., which infects a wide range of mammalian species including humans, is a significant cause of gastroenteritis among young children [5,6]. Children living in resource-poor settings in sub-Saharan Africa and South Asia are particularly at risk of cryptosporidiosis [7].

*Cryptosporidium* spp. are transmitted through the fecal-oral route via the consumption of water or food contaminated with fecal matter, as well as by direct contact with infected individuals or animals [5]. It is highly infectious, and its transmission stage (the oocyst) is resistant to the disinfectants routinely used in the drinking water industry [8]. Globally, childhood cryptosporidiosis is estimated to cause approximately 44.8 million diarrheal episodes and 48,300 deaths annually, with the majority occurring in Africa [9]. Its burden is particularly high in sub-Saharan Africa, including Ethiopia, where *Cryptosporidium* infection is associated with prolonged diarrhea and a two- to three-fold increase in mortality among children with diarrhea [4,9].

Several factors may have a role in contributing to the high prevalence of *Cryptosporidium* infection, including the presence of infected individuals within households, contact with animals, open defecation practices, and inadequate breastfeeding [10,11]. However, the true magnitude of *Cryptosporidium* infections in low-income countries is likely underestimated due to the lack of systematic diagnostic testing for diarrheal disease etiologies and the widespread reliance on less-sensitive microscopic detection methods during routine clinical practice. Traditional diagnostic approaches, including microscopy with modified Ziehl-Neelsen staining, have limited sensitivity and require specialized expertise. Advanced techniques, like antigen-detection assays and polymerase chain reaction (PCR), offer improved sensitivity, but are not widely implemented in low-resource settings due to cost and technical constraints. This diagnostic gap contributes to an underestimation of the true prevalence and burden of *Cryptosporidium* infections in these regions [10,12].

Despite diarrhea being a significant health issue in Ethiopia [13,14], studies specifically focusing on *Cryptosporidium* infection remain limited. Existing studies on *Cryptosporidium* infection in Ethiopia show prevalence rates varying from 1% to 26% [15,16]. Most of this research has concentrated on individuals living with HIV, with relatively few studies involving school-aged or diarrheic children [16,17]. In addition, most of the previous studies used microscopic examination of stool smear using modified Ziehl-Neelsen staining, a method with a sensitivity of 55–75% and a specificity of 96–100% [10,18]. In contrast, stool samples stained with auramine-phenol (AP) and examined using light-emitting diode (LED) fluorescence microscopy have demonstrated superior diagnostic performance, with a sensitivity of 88% and a specificity of 99% [19]. Notably, there are no data on *Cryptosporidium* infection among young children in Eastern Ethiopia. Therefore, the present study aimed to assess the prevalence and factors associated with *Cryptosporidium* infection among children under five years of age with diarrhea in Eastern Ethiopia, using the light-emitting diode- auramine-phenol (LED-AP) method of diagnosis.

## Methods

### Ethics statement

The study protocol was reviewed and approved by the College of Health and Medical Sciences Institutional Health Research Ethics Review Committee (IHRERC) of Haramaya University (Ref. No.; IHRERC/100/2022), and the Armauer Hansen Research Institute (AHRI)/ All African Leprosy and Tuberculosis Rehabilitation and Training Center (ALERT) Ethics Review Committee (AAERC) (Protocol number; PO-33–22). The study participants were informed of their rights to withdraw from the study at any time. Before commencement of data collection, informed voluntary written and signed consents was obtained from each child’s parent or guardian for their own participation in the questionnaire and on behalf of their child for the provision of a stool sample. Moreover, all data collection procedures in this study followed the ethical guidelines of the Declaration of Helsinki, ensuring participant safety and integrity

### Study area

**Fig 1:**
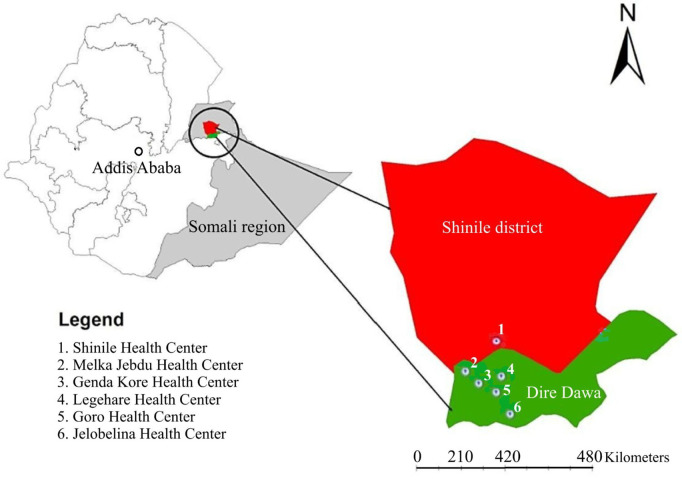
Map of the study area in Eastern Ethiopia. The map was constructed in ArcGIS 10.8.2 using district boundaries, https://gadm.org/download_country.html, and health facilities collected during the study. Data available under CC_BY 4.0 (Creative Commons Attribution, https://creativecommons.org/) license.

The study was conducted in six health centers, one from Shinile and five from the Dire Dawa Administration (DDA) (**Fig 1**). The study settings have two main seasons, a wet season from April to September and a dry season from October to March. Shinile is one of the woredas (districts) in the Somali region of Ethiopia, located at 9°40′N latitude and 41°50′E longitude. Its total population in 2022 was 70,181, of which 7,271 were children under five years of age (Shinile district report, 2022). The district has three health centers, but only one was fully operational and included in this study. Dire Dawa is located at 9°36′N latitude and 41°52′E longitude, approximately 515 km East of the capital, Addis Ababa, and the total population of DDA in 2022 was projected to be 535,684. Among the population, 192,106 individuals resided in rural kebeles, and 52,974 were children under five years of age [20]. DDA has two public hospitals and 14 health centers (Dire Dawa administration Regional Health Bureau report, 2022). For this study, five health centers (Gendakore, Goro, Jelobellina, Lagahare, and Melkajebdu) were randomly selected using a lottery method.

### Study design and populations

This was a cross-sectional study conducted among diarrheic children under five years of age attending the selected health centers. The study was conducted from November 2022 to October 2023, covering all months of the year to assess seasonal variations of infection. The caregivers who consented for their children’s participation and diarrheic children who could provide a stool specimen, and a completed questionnaire were eligible to enroll in the study. Children admitted as inpatients were excluded, as we were interested in factors for infection in the childrens’ home environments and wished to avoid confounding with nosocomial infectons.

### Sample size determination and sampling technique

The target sample size of the study was determined using single population proportion formula n = Z^2^pq/d^2^ where n = sample size, Z = level of confidence interval (95%), p = prevalence of *Cryptosporidium* infection (13%) from a multi-country study in sub-Saharan Africa [21] and q = proportion of those negative for disease of interest = 1-p and d: margin of error (set at 2.5%). A 15% non-response rate was included, assuming some children might be unable or unwilling to provide stool samples. The final sample size was then proportionally allocated to the selected health centers based on patient attendance data from the previous year. A systematic random sampling technique was employed to enroll study participants during daytime hours at the respective health centers based on their diarrheic patient caseload.

### Measurements

Diarrhea was defined as the passage of three or more loose or watery stools or at least one episode of dysentery within a 24-hour period [22]. Dysentery was defined as the occurrence of at least one loose stool containing visible blood within a day. A caregiver was defined as the parent or guardian who brought the child to the selected health centers. *Cryptosporidium* infection was defined as the detection of *Cryptosporidium* oocysts by LED-AP fluorescence microscopy. Socio-demographic characteristics, such as age, sex, caregiver’s education, caregiver’s occupation (the work he/she spent most of his/her time on), marital status, number of rooms for living, birth order of child, family size, presence of animals and availability of toilet, were collected from caregivers using a structured questionnaire.

Moreover, information on handwashing, history of contact with animals (defined as having close contact with a domestic animal or their excrement in their playground area), history of contact with diarrheic individuals in the household, exclusively breastfed, feed preparation, treatment of drinking water, use of toilet paper, having fever, duration of diarrhea, and previous episodes of diarrhea were collected in the same way as above. For children younger than six months, exclusive breastfeeding status was described as the child having been exclusively breastfed up to the time of data collection. Caregivers of children older than six months were asked about past breastfeeding practices to ascertain whether exclusive breastfeeding had occurred during the first six months of the child’s life.

### Data and sample collection

Face-to-face interviews were conducted using an epidemiological questionnaire prepared in English, which was then translated into local languages (Afan Oromo, Somali, and Amharic) and translated back into English to ensure consistency. The questionnaire was pretested with 5% of the study participants at Addis Ketema Health Center in Dire Dawa. The feedback gathered from this pretest was used to make necessary modifications to the questionnaire. Data collectors were trained in basic interviewing techniques. The study investigator checked the data daily for completeness. Approximately 5–10 g of fresh fecal specimens was collected into clean stool containers after providing instructions to caregivers on proper collection procedures. After collection, the stool specimens were examined using LED-AP microscopy at the study sites.

### Detection of *Cryptosporidium* by LED-AP microscopy

A thin fecal smear was prepared immediately after stool sample collection and stained using the AP staining technique [19]. Briefly, the smear was fixed and flooded with AP stain solution for 15 min, then washed with distilled water and decolorized using acid ethanol solution for 2 min. Finally, it was counterstained with potassium permanganate solution for 2 min to reduce background fluorescence. *Cryptosporidium* oocysts, measuring 4–6 µm in diameter, appeared as ring- or doughnut-shaped structures with a greenish-yellow fluorescence against a dark background.

Each stained slide was examined by medical laboratory technologists trained in the method using LED-AP fluorescence microscopy in each study health center. All slides were then stored in a closed slide box and transported to the microbiology laboratory at Haramaya University College of Health and Medical Sciences, where they were blindly re-examined for validation by independently trained laboratory personnel. In case of discrepant results, the study investigator re-examined the slides. The presence or absence of *Cryptosporidium* was determined using a 20 × objective lens with a 10 × eyepiece. Presumptive objects were further examined under 40× and 100 × objectives. Additionally, the *Cryptosporidium* oocyst load in each stool specimen was estimated using a semi-quantitative method. A minimum of 10 fields were examined under 200 × magnification, and the average number of oocysts per field was categorized as **+** (1–9 oocysts), **++** (10–50 oocysts) and **+++** (> 50 oocysts) as described elsewhere [23].

### Statistical analysis

Data were entered into Epidata version 3.1 and exported to Stata version 17 (StataCorp LLC, 2021) for cleaning and analysis. Descriptive and inferential statistics were used to summarize and interpret the data. Bivariate and multivariate robust Poisson regression models were employed to assess the association between *Cryptosporidium* infection and potential associated factors. The Hosmer-Lemeshow test was used to evaluate the goodness-of-fit of each multivariate regression model, and multicollinearity among predictor variables was also assessed using Variance inflation factor. Variables with a *P*-value of less than 0.25 in the bivariate analysis were included in the multivariate model. The strength of associations between *Cryptosporidium* infection and associated factors was estimated using prevalence ratios (PR) with 95% confidence intervals (CI). An association was considered statistically significant at p-value ≤ 0.05 in the multivariate Poisson regression model with robust variance estimation.

## Results

### Characteristics of study participants

A total of 756 children under five years of age, along with their caregivers, participated in this study. The male to female ratio was 1:0.8. The majority were from Lagahare and Goro health centers in Dire Dawa. A total of 548 (72.5%) and 176 (23.3%) of caregivers were the children’s mothers and fathers, respectively. Three hundred and two (40.0%) of the caregivers had no formal education. The majority (92.0%) reported having a latrine, of which 77.0% had a simple pit latrine (unimproved latrine). Around 30.0% of households owned at least one species of livestock. Among those who owned livestock, the majority (87.0%) had goats, while 51.0% had cattle. Moreover, 29.0% of households also had pet animals (dogs and cats). The majority (55.0%) of the children included were males. The children’s ages ranged from one to fifty-nine months (mean± SD = 20.8 ± 13.4; median = 16 months), and approximately 70% were under two years old (**Table 1**).

**Table 1:** Sociodemographic characteristics of study participants and prevalence of Cryptosporidium infection in Eastern Ethiopia (n = 756).

Variables	Categories	Total	*Cryptosporidium* Positives	χ² P-value
		Number	Number (%)	
Distribution by Health centers	Lagahare	215	33 (15.3)	0.55
	Goro	185	27 (14.6)	
	Gendakore	164	23 (16.3)	
	Melkajebdu	69	13 (18.8)	
	Jelobelina	32	8 (25.0)	
	Shinile	91	11 (12.1)	
Caregivers’ relation to child	Father	176	26 (14.8)	0.82
	Mother	548	84 (15.3)	
	Guardian	32	5 (15.6)	
Age of caregivers (years)	<26	150	23 (15.3)	0.26
	26–35	497	81 (16.3)	
	>35	109	11 (10.1)	
Sex of Caregivers	Male	189	28 (14.8)	0.86
	Female	567	87 (15.3)	
Marital status of Caregivers	Not married	47	11 (23.4)	0.11
	Married	709	104 (14.7)	
Caregivers’ Educational level	No formal education	302	64 (21.2)	0.01
	Grade 1–4	58	12 (20.7)	
	Grade 5–8	139	18 (13.0)	
	Grade 9–12	119	10 (8.4)	
	Diploma and above	138	11 (8.0)	
Occupation of Caregivers	Farmer	180	48 (26.7)	<0.001
	Trader	87	14 (16.1)	
	Employer	194	20 (10.3)	
	Housewife	295	33 (11.2)	
Family size	2–3	208	20 (9.6)	0.03
	4–5	402	68 (16.9)	
	>5	146	27 (18.5)	
Number of children under five years of age in family	1	454	61 (13.4)	0.22
	≥2	302	54 (18.0)	
Availability of Latrine	Yes	699	95 (13.6)	<0.001
	No	57	20 (35.0)	
Types of latrines of household	Unimproved	580	81 (14.0)	0.69
	improved	119	14 (11.8)	
Owner of latrine	Private	286	37 (13.0)	0.67
	Communal	413	58 (14.0)	
Household have Livestock	Yes	227	43 (19.0)	0.06
	No	529	72 (13.6)	
Household have pets	Yes	222	38 (17.1)	0.35
	No	534	77 (14.4)	
Child sex	Male	415	61 (14.7)	0.66
	Female	341	54 (15.8)	
Age of child in months	≤12	293	40 (13.7)	0.76
	13–24	240	40 (16.7)	
	25–36	127	19 (15.0)	
	>36 < 60	96	16 (16.7)	

χ²: Chi-Square, %: percentage

### Prevalence of *Cryptosporidium* infections

Overall, 15.2% (115/756; 95% CI: 12.7–17.9) of the children enrolled in the study were positive for *Cryptosporidium* by LED-AP microscopy. Of the 115 *Cryptosporidium*-positive samples, 41 (35.6%), 46 (40%), and 28 (24.4%) had oocyst loads categorized as 1 + , 2 + , and 3 + , respectively. The proportion of *Cryptosporidium*-positive samples was similar in each of the study health centers, ranging from 12.1% to 18.9%; in Jelobelina health center the proportion was higher (25.0%), but this was not statistically significant (**Table 1**).

**Fig 2:**
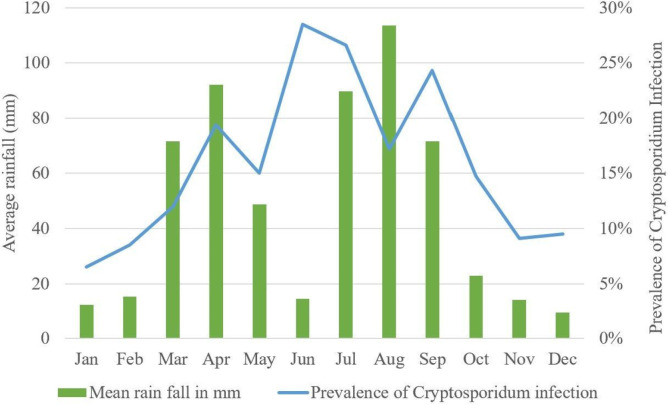
Association between prevalence of Cryptosporidium infection among children under five years old with diarrhea and average rainfall (mm) of the study area.

There was no significantly higher occurrence of *Cryptosporidium* in any of the four age groups being compared (**Table 1**). There was a significantly higher occurrence of *Cryptosporidium* infection among children who were not exclusively breastfed. The highest prevalence of *Cryptosporidium* infection was detected in June (28.5%), followed by July (26.6%) and September (24.3%) (**Fig 2**). Moreover, *Cryptosporidium* infection was detected in 24.2% of children with diarrhea lasting more than three days before seeking medical care and 18.0% of those presenting with fever.

### Factors associated with *Cryptosporidium* infection

Results obtained in our bivariate and multivariable analyses are presented in Supporting information (S1 Table) and **Table 2**, respectively. The variables “age” and “season” were included in the final model regardless of the p-values obtained in the bivariate analysis, assuming them to be potential confounders for many of the factors.

**Table 2:** Factors associated with Cryptosporidium infections among diarrheic children under five years of age attending health centers in Eastern Ethiopia, 2023 (n = 756).

Variables		Total	*Cryptosporidium* Positive	Bivariate analysis	Multivariate analysis	
		Number	Number (%)	CPR (95% CI)	APR (95% CI)	*P*-value
Season	Dry	364	35 (9.6)	1	1	
	Wet	392	80 (20.4)	2.1 (1.5-3.1)	1.9 (1.3-2.7)	0.01
Caregivers’ educational level	No formal education	302	64 (21.2)	2.7 (1.4-4.9)	2.4 (1.1-5.3)	0.03
	Grade 1–4	58	12 (20.7)	2.6 (1.2-5.5)	2.1 (0.9-5.2)	0.09
	Grade 5–8	139	18 (13.0)	1.6 (0.8-3.3)	2.0 (0.9-4.6)	0.08
	Grade 9–12	119	10 (8.4)	1.1 (0.5-2.4)	1.2 (0.5-3.0)	0.72
	Diploma and above	138	11 (8.0)	1	1	
Occupation of caregivers	Farmer	180	48 (26.7)	1	1	
	Trader	87	14 (16.1)	0.6 (0.4-1.0)	1.4 (0.8-2.3)	0.25
	Employer	194	20 (10.3)	0.4 (0.2-0.6)	1.5 (0.7-2.9)	0.29
	Housewife	295	33 (11.2)	0.4 (0.3-0.6)	0.9 (0.6-1.3)	0.46
Family size	2-3	208	20 (9.6)	1	1	
	4-5	402	68 (17.0)	1.8 (1.1-2.8)	0.9 (0.5-1.6)	0.69
	Greater than 5	146	27 (18.5)	1.9 (1.1-3.3)	0.6 (0.3-1.1)	0.10
Diarrhea in the household	Yes	266	74 (27.8)	3.3 (2.3-4.7)	2.0 (1.2-3.1)	0.01
	No	490	41 (8.4)	1	1	
Contact with diarrheic individuals	Yes	183	62 (33.9)	3.7 (2.6-5.1)	1.7 (1.1-2.5)	0.02
	No	573	53 (9.2)	1	1	
Household owns Livestock	Yes	227	43 (19.0)	1.4 (1.0-2.00)	1.2 (0.9-1.6)	0.32
	No	529	72 (13.6)	1	1	
Contact with animals	Yes	165	28 (17.0)	2.5 (1.8-3.53)	0.8 (.6 1.1)	0.14
	No	591	87 (14.7)	I	1	
Treating drinking water	Yes	150	16 (10.7)	1	1	
	No	606	99 (16.3)	1.5 (0.9-2.5)	1.1 (0.6-1.9)	0.72
Availability of latrine	Yes	699	95 (13.6)	1	1	
	No	57	20 (35.1)	2.6 (1.7-3.9)	1.3 (0.9-2.0)	0.20
Washing of hand after toilet use	Yes	557	50 (9.0)	1	1	
	No	199	65 (32.7)	3.6 (2.6-5.1)	2.8 (1.7-4.5)	0.01
Using of toilet paper	Yes	309	71 (24.0)	2.3 (1.6-3.3)	1.6 (1.1-2.3)	0.01
	No	447	44 (9.8)	1	1	
Child’s age (Months)	≤12	293	40 (13.7)	1	1	
	13–24	240	40 (16.7)	1.2 (0.8-1.8)	1.1 (0.7-1.6)	0.69
	25–36	127	19 (15.0)	1.1 (0.6-1.9)	1.0 (0.7-1.6)	0.87
	>36	96	16 (16.7)	1.2 (0.8-1.9)	1.1 (0.7-1.7)	0.17
Birth order of child	First/only child	235	21 (9.0)	1	1	
	Second	254	35 (13.8)	1.5 (0.9-2.5)	1.6 (0.9-3.0)	0.14
	Third	165	36 (21.8)	2.44 (1.4-4.0)	1.3 (0.7-2.6)	0.32
	Above third	102	23 (22.5)	2.5 (1.5-4.4)	2.0 (1.1-3.8)	0.03
Exclusively breastfeed	Yes	527	53 (10.1)	1	1	
	No	229	62 (27.1)	2.7 (1.9-3.8)	1.8 (1.3-2.6)	0.01
hand Washing after helping child defecate	Always	585	73 (12.5)	1	1	
	Sometimes	116	27 (23.3)	1.9 (1.3-2.8)	0.9 (0.6-1.4)	0.67
	Rarely	55	15 (27.3)	2.2 (1.4-3.5)	1.1 (0.6-1.9)	0.83
Cleaning child after defecation	Always	579	71 (12.3)	1	1	
	Sometimes	131	31 (23.7)	1.9 (1.3-2.8)	1.0 (0.6-1.6)	0.84
	Rarely	46	13 (28.3)	2.3 (1.4-3.8)	0.8 (0.4-1.5)	0.53
Washing hand before feeding	Always	574	72 (12.5)	1	1	
	Sometimes	139	31 (22.3)	1.8 (1.2-2.6)	1.1 (0.7-1.8)	0.78
	Rarely	43	12 (28.0)	2.2 (1.3-3.8)	0.8 (0.4-1.4)	0.37
Using of soap for washing hand	Yes	485	49 (10.1)	1	1	
	No	271	66 (24.4)	2.4 (1.7-3.4)	1.3 (0.9-1.9)	0.16

APR: Adjusted Prevalence Ratio, CI: Confidence Intervals, CPR: Crude Prevalence Ratio and %: percentage.

In the final model, *Cryptosporidium* infections were nearly twice as likely to occur during the wet season than the dry season. Children whose caregivers had no formal education were 2.4 times more likely to be infected with *Cryptosporidium* than children whose caregivers had a diploma or higher educational status. Children living in households with a family member reporting diarrhea in the two weeks prior to sampling were two times more likely to be infected with *Cryptosporidium* compared to children in households without recent diarrhea cases. Similarly, children who had contact with individuals experiencing diarrhea were 1.7 times more likely to be infected with *Cryptosporidium* compared to their counterparts (**Table 2**).

Caregivers that did not wash their hands after toileting were nearly three times more likely to have children infected with *Cryptosporidium* than those who practiced handwashing. Similarly, Caregivers who used toilet paper were 1.6 times more likely to have children infected with *Cryptosporidium* than those did not use it. This means using toilet paper alone is not a hygienic intervention unless paired with proper handwashing, safe disposal, and clean sanitation practices. Children with three or more older siblings were two times more likely to be infected with *Cryptosporidium* compared to those with no older siblings. Children who were not exclusively breastfed were 1.8 times more likely to be infected than their exclusively breastfed counterparts (**Table 2**).

## Discussion

This study found that the prevalence of *Cryptosporidium* infection among diarrheic children under five years old seeking healthcare in Eastern Ethiopia was 15.2% (95% CI: 12.7–17.9). Season, caregivers’ educational status, childbirth order, exclusive breastfeeding, household history of diarrhea, contact with diarrheic individuals, handwashing after toileting, and the use of toilet paper were factors significantly associated with an increased risk of *Cryptosporidium* infection.

The prevalence of *Cryptosporidium* infection in the current study is consistent with some of those reported in similar studies conducted among children with diarrhea attending healthcare facilities in Ethiopia and other sub-Saharan African countries (S1 Table). *Cryptosporidium* prevalence rates of 12.8% was documented in diarrheic pediatric population in Bahir Dar, Ethiopia [24], 12.9% in Gabon [25], 13.9% in Mozambique [26], and 15.0% and 15.6% in Tanzania [21,27]. However, the prevalence found in this study is higher than previous reports from Ethiopia such as 9.0% in Jimma [23]. It is also higher than the prevalence reported in a sub-study conducted among 58 symptomatic children under three years of age attending health facilities in Mekelle, which found a prevalence of 5.2% [28]. Similarly, the prevalence observed in this study is higher than reports from other parts of Africa which reported 11.0% in Ghana and 12.0% in Madagascar [21] 8.4% and 11.0% in Kenya [29,30], 9.1% in Malawi [31], 3.4 and 11.0% in Mozambique [32,33], 4.0% in Nigeria [34] and 6.0% in South Africa [35]. In contrast, the prevalence found in this study was lower than those reported in some other African studies (S2 Table) including rates of 30.0% in Angola [36], 20.5% in Botswana [6], 21.0% in Gabon [21], 18.4% in Guinea-Bissau [37], 23.5% and 32.0% in Kenya [17,38], 22.8% and 23.1% in Nigeria [39,40] and 27.1% in Sudan [41]. These discrepant figures may be due to differences in local environmental conditions, seasonal variations, nutritional habits, personal hygiene practices, sanitation, access to safe drinking water, sewage water management, and methodological factors such as sample size, sampling techniques and diagnostic procedures. Moreover, the higher prevalence observed in this study compared to some previous studies that used conventional microscopy examination may be attributed to the use of more sensitive and specific LED-AP fluorescence microscopy in the current study.

In this study, there was a statistically significant association between season and the prevalence of *Cryptosporidium* infection, which was nearly two times more likely to occur during the wet season than during the dry season, with the highest prevalence rate (28.6%) being detected in June. Similar findings have been reported in previous studies. For instance, cryptosporidiosis cases peaked during the hottest months in Pakistan [42], and during the period April-June in Kenya [30]. These findings may be due to the fact that children tend to gather and play together more frequently during this season, thereby increasing their contact with surface waters potentially contaminated with *Cryptosporidium* oocysts. Additionally, in the study area, there is a common practice of storing water for future use. Furthermore, the area is prone to flooding during the rainy season, which may contaminate rivers, streams, and stored water sources with fecal matter. These water sources are used for cattle feeding, irrigation, and drinking, increasing the risk of *Cryptosporidium* infection among residents.

Caregivers’ educational status was significantly associated with *Cryptosporidium* infection in children, with a higher likelihood of infection among children whose caregivers had no formal education. Similarly, previous studies conducted in Cameroon [43], Ghana [44] and Mozambique [26] have found that a lack of secondary or higher education was associated with an increased risk of *Cryptosporidium* infection. Another study conducted in Mozambique reported that children of illiterate caregivers were more likely to be infected with *Cryptosporidium* [45]. This is likely because education is associated with improved hygiene practices, sanitation conditions, feeding habits, handwashing behaviors, and breastfeeding duration. Moreover, more educated parents may have higher incomes, enabling them to afford hygiene products such as soap and bottled water for drinking and food preparation for their children.

The current study showed that children with three or more older siblings were two times more likely to be infected with *Cryptosporidium* compared to those with no older siblings. This finding is consistent with a study conducted in India reporting the presence of an older sibling in the household as a significant risk factor for cryptosporidiosis [46]. Additionally, an increase in the number of siblings living at home in Peru has been associated with higher odds of *Cryptosporidium* infection [47]. This may be because overcrowding conditions facilitate closer and more frequent personal contacts, enhancing the transmission risk of *Cryptosporidium* through the fecal-oral route [48]. Furthermore, older siblings, who may have lower hygiene standards than adults, are often involved in the care of younger siblings, potentially increasing the risk of infection.

Breast milk provides effective passive immunity to newborns against a wide range of enteric pathogens [49]. Children who were not exclusively breastfed were 1.6 times more likely to develop *Cryptosporidium* infection than those who were exclusively breastfed. This finding is consistent with a study conducted in south east of Iran that reported breastfed infants were less susceptible to *Cryptosporidium* infection [50]. Similarly, a study in Cameroon found a higher prevalence of *Cryptosporidium* infection among children who were not exclusively breastfed compared to those who were [43]. Other studies have also reported that non-exclusively breastfed children are more likely to develop diarrhea [51,52]. This protective effect of breastfeeding is attributed to the presence of secretory immunoglobulin A in breast milk, which helps shield children from mucosal pathogens. Additionally, breast milk contains other protective molecules such as IgG, IgM, IgD, lactoferrin, lactoperoxidase, and various leukocytes that safeguard the mucous membrane against gastrointestinal pathogens [53]. Moreover, children who are not exclusively breastfed may be more exposed to *Cryptosporidium* through contaminated food and drinks.

We found that diarrhea in the household and contact with diarrheic individuals were significant risk factors for *Cryptosporidium* infection among diarrheic children (p-value < 0.05). Similarly, previous studies have indicated that household diarrhea is one of the most relevant risk factors associated with *Cryptosporidium* infection in low- and middle-income countries [11]. Additionally, contact with a person with diarrhea has been identified as a risk factor for *Cryptosporidium* infection. This implies that close person-to-person contact is a major transmission route for *Cryptosporidium*, consistent with findings from other studies [21,54].

Furthermore, this study showed that not washing hands after using the toilet and using toilet paper or were significant risk factors for *Cryptosporidium* infection in children. This may be because individuals who use toilet paper perceive themselves as clean, and therefore often neglect thorough washing of their hands. This can lead to contamination of food, surfaces, or direct contact with children, facilitating fecal-oral transmission of *Cryptosporidium.* The used toilet paper may also contaminate the environment around the toilet, and children may acquire the infection from that environment or from family members. Supporting this, a study found that infrequent handwashing practices were an independent predictor of increased diarrheal morbidity [55]. Taken together, these findings highlight the importance of case contact in transmitting *Cryptosporidium*, similar to other fecal-orally infectious diseases, and emphasize the role of personal hygiene such as proper handwashing and child hygiene in reducing *Cryptosporidium* transmission.

In this study, *Cryptosporidium* infection was more prevalent in children who had diarrhea for more than three days before the study (p-value < 0.05). This finding is consistent with studies from other sub-Saharan African countries, which have shown that *Cryptosporidium* infection is associated with prolonged or persistent diarrhea [4,9,29]. Moreover, fever was commonly observed in children with *Cryptosporidium* infection in this study. However, other studies have reported no significant association between fever and *Cryptosporidium* infection [48,56].

In the present study, age and gender of the child, treatment of drinking water, availability of a latrine, and handwashing before feeding and after helping a child defecate showed no significant association with *Cryptosporidium* infection (p > 0.05). Previous studies have also indicated that the prevalence of cryptosporidiosis was not statistically associated with age [43] and gender, with a p-value of >0.05 [42,57]. This may be because children are likely exposed to the same risk factors, regardless of their sex [58]. However, a study conducted in Tigray (north Ethiopia) showed that *Cryptosporidium* infection was related to age, with higher prevalence in younger age groups (≤ 15 years), and to sex, with a higher prevalence in females [28]. This discrepancy may be due to the broader age range in the Tigray study, which included individuals aged 4–80 years, whereas the current study focused on children aged 1–59 months. Unlike young children, adult females and males may not be equally exposed to the same risk factors for *Cryptosporidium* infection, and there may also be differences in their immune responses.

In contrast to the well-established role of animals in the transmission of cryptosporidiosis in some settings [59], as well as findings from a systematic review and meta-analysis in low- and middle-income countries [11] and in Ethiopia [16], which indicated that animal contact was significantly associated with *Cryptosporidium* infection, this study found that the presence and contact with animals were not significantly associated with cryptosporidiosis among children. This finding is consistent with a previous study in Ethiopia and elsewhere [60,61]. This may be due to the fact that zoonotic transmission of *Cryptosporidium* occurs relatively rarely in Sub-Saharan Africa compared with in developed countries like in Europe, predominantly due to the *Cryptosporidium* species distribution being very different [62,63]. In our study also, where livestock ownership was not associated with *Cryptosporidium* infection, anthroponotic species, particularly *C. hominis*, predominated in the study area [64], as has previously been observed in other urban and semi-urban settings of developing countries [62]. In addition, among the of *Cryptosporidium* species detected from livestock fecal samples investigated during the study period, only *C. ubiquitum*, *C. xiaoi* and *C. ryanae* were identified, of which only *C. ubiquitum* is considered to have zoonotic potential [64].

This study has strengths, such as utilizing a more sensitive and specific laboratory test, AP fluorescence microscopy, to diagnose *Cryptosporidium* infection, unlike most previous studies that relied on the modified Ziehl-Neelsen staining method. As a result, this study provides a relatively more accurate estimate of the infection burden. Additionally, data were collected over a one-year period, which is important for assessing seasonal variations in *Cryptosporidium* infection among diarrheic children under five in eastern Ethiopia. The study is further strengthened by the molecular characterization of *Cryptosporidium* species from both children and livestock [64]. It also has some limitations, such as the cross-sectional design, which assesses variables at a single point in time, making it challenging to infer cause-and-effect relationships. Additionally, there may be recall bias, as caregivers may provide incorrectly recalled information about symptoms or risk factors. Only a single stool sample was tested per child, potentially leading to false negatives, as *Cryptosporidium* oocysts may shed inconsistently. Therefore, the findings of this study should be interpreted with caution.

## Conclusions

*Cryptosporidium* spp. infection was found to be highly prevalent in diarrheic children under five years of age seeking healthcare in Eastern Ethiopia. Several factors were significantly associated with the infection, including hygiene parameters in the child’s environment, exclusive breastfeeding status, diarrhea in the household, seasonal effects, and caretakers’ educational status. Therefore, raising community awareness about the seasonality of disease, the importance of exclusive breast feeding, and the relevance of proximity and person to person close contact for transmission of *Cryptosporidium* infection is recommended. Molecular-based studies that investigate the frequency and diversity of *Cryptosporidium* species and genotypes would also be valuable for better understanding the sources of transmission in Ethiopia.

## Supporting information

S1 Table Binary robust Poison regression analysis to select candidate variables for multiple robust Poison regression. (DOCX)

S2 Table Studies reporting prevalence of *Cryptosporidium* infection among diarrheic children under five years of age in Sub-Saharan Africa countries. (DOCX)

S1 Data Study data. (XLSX)
