# Supplementary material for: Cryptosporidium infection and associated factors among diarrheic children under five years of age in Eastern Ethiopia
Source: PLoS Negl Trop Dis. 2025 Aug 5;19(8):e0013386. doi: 10.1371/journal.pntd.0013386 (PMC12349710; doi:10.1371/journal.pntd.0013386)
Supplement: S1 Table — (DOCX) [file pntd.0013386.s001.docx]

S1 Table: Binary robust Poisson regression analysis to select candidate variables for multiple robust Poisson regression

| Variables | | Presence of *Cryptosporidium* infections | | | Binary robust Poisson regression | |
| --- | --- | --- | --- | --- | --- | --- |
|  |  | Yes | No | Total | CPR (95% CI) | p-value |
| Season | Dry | 35 | 329 | 364 | 1 |  |
|  | Wet | 80 | 312 | 392 | 2.1 (1.50-3.10) | <0.001 |
| Age of caregivers (years) | <26 | 23 | 127 | 150 | I |  |
|  | 26-35 | 81 | 416 | 497 | 1.06 (0.69-1.63) | 0.78 |
|  | >35 | 11 | 98 | 109 | 0.66 (0.34-1.30) | 0.23 |
| Sex of caregivers | Male | 28 | 161 | 189 | I |  |
|  | Female | 87 | 480 | 567 | 1.04 (0.70-1.53) | 0.86 |
| Caregivers’ relation to child | Father | 26 | 150 | 176 | I |  |
|  | Mother | 84 | 464 | 548 | 1.04 (0.69-1.56) | 0.86 |
|  | Guardian | 5 | 27 | 32 | 1.06 (0.44-2.55) | 0.90 |
| Caregivers’ marital status | Not Married | 11 | 36 | 47 | I |  |
|  | Married | 104 | 605 | 709 | 0.63 (0.36-1.08) | 0.94 |
| Caregivers’ educational status | No formal education | 64 | 238 | 302 | 2.70 (1.40-4.9) | 0.01 |
|  | Grade 1-4 | 12 | 46 | 58 | 2.60 (1.20-5.50) | 0.01 |
|  | Grade 5-8 | 18 | 121 | 139 | 1.60 (0.80-3.30) | 0.18 |
|  | Grade 9-12 | 10 | 109 | 119 | 1.10 (0.50-2.40) | 0.90 |
|  | Diploma and above | 11 | 127 | 138 | 1 |  |
| Caregivers’ occupation | Farmer | 48 | 132 | 180 | I |  |
|  | Trader | 14 | 73 | 87 | 0.60 (0.35-1.03) | 0.06 |
|  | Employer | 20 | 174 | 194 | 0.39 (0.24-0.62) | <0.001 |
|  | Housewife | 33 | 262 | 295 | 0.42 (0.28-0.63) | <0.001 |
| Caregivers’ zoonotic diseases knowledge | Yes | 9 | 72 | 81 | I |  |
|  | No | 106 | 569 | 675 | 1.41 (0.74-2.68) | 0.29 |
| Number of rooms for living | 1 | 74 | 326 | 400 | 2.10 (1.14-3.80) | 0.26 |
|  | 2 | 30 | 202 | 232 | 1.46 (0.76-2.81) | 0.47 |
|  | ≥3 | 11 | 113 | 124 | I |  |
| Number of rooms for sleeping | 1 | 103 | 558 | 661 | 1.23 (0.71-2.15) | 0.46 |
|  | ≤2 | 12 | 83 | 95 | I |  |
| Family size | 2-3 | 20 | 188 | 208 | I |  |
|  | 4-5 | 68 | 334 | 402 | 1.76 (1.10-2.81) | 0.02 |
|  | >5 | 27 | 119 | 146 | 1.92 (1.12-3.30) | 0.02 |
| Diarrhea in the household | Yes | 74 | 192 | 266 | 3.32 (2.34-4.72) | <0.001 |
|  | No | 41 | 449 | 490 | I |  |
| Contact with diarrheic individuals | Yes | 62 | 121 | 183 | 3.66 (2.64-5.08) | <0.001 |
|  | No | 53 | 520 | 573 | I |  |
| Household owns livestock | Yes | 43 | 184 | 227 | 1.39 (0.99-1.96) | 0.06 |
|  | No | 72 | 457 | 529 | I |  |
| Household owns pets | Yes | 38 | 184 | 222 | 1.18 (0.83-1.69) | 0.35 |
|  | No | 77 | 457 | 534 | I |  |
| Neighbors had animals | Yes | 26 | 113 | 139 | 1.29 (0.87-1.93) | 0.20 |
|  | No | 89 | 528 | 617 | I |  |
| Contact with animals | Yes | 28 | 137 | 165 | 2.54 (1.83-3.54) | 0.01 |
|  | No | 87 | 504 | 591 | I |  |
| Household drinking water treatment | Yes | 16 | 134 | 150 | I |  |
|  | No | 99 | 507 | 606 | 1.53 (0.93-2.52) | 0.09 |
| Availability of latrine | Yes | 50 | 604 | 699 | I |  |
|  | No | 20 | 37 | 57 | 2.58 (1.730-3.850) | <0.001 |
| Washing of hand after toilet use | Yes | 50 | 507 | 557 | I |  |
|  | No | 65 | 134 | 199 | 3.64 (2.61-5.07) | <0.001 |
| Using of toilet paper/soft tissue | Yes | 71 | 238 | 309 | 2.33 (1.65-3.30) | <0.001 |
|  | No | 44 | 403 | 447 | 2.78 (1.83-4.22) | <0.001 |
| Sex of child | Male | 61 | 354 | 415 | I |  |
|  | Female | 54 | 287 | 341 | 1.08 (0.77-1.51) | 0.66 |
| Age of child in months | ≤12 | 40 | 253 | 293 | I |  |
|  | 13-24 | 40 | 200 | 240 | 1.22 (0.82-1.83) | 0.33 |
|  | 25-36 | 19 | 108 | 127 | 1.10 (0.66-1.82) | 0.72 |
|  | >36 | 16 | 80 | 96 | 1.22 (0.72-2.10) | 0.46 |
| Birth order of child | First/only child | 21 | 214 | 235 | I |  |
|  | Second | 35 | 219 | 254 | 1.54 (0.92-2.57) | 0.09 |
|  | Three | 36 | 129 | 165 | 2.44 (1.48-4.03) | <0.001 |
|  | Fourth and above | 23 | 79 | 102 | 2.52 (1.46-4.35) | 0.01 |
| Exclusively breastfed | Yes | 53 | 474 | 527 | I |  |
|  | No | 62 | 167 | 229 | 2.69 (1.93-3.75) | <0.001 |
| Washing hands after helping child defecate | Always | 73 | 512 | 585 | I |  |
|  | Sometimes | 27 | 89 | 116 | 1.87 (1.26-2.77) | 0.01 |
|  | Rarely | 15 | 40 | 55 | 2.20 (1.35-3.54) | 0.01 |
| Cleaning child after defecation | Always | 71 | 508 | 579 | I |  |
|  | Sometimes | 31 | 100 | 131 | 1.93 (1.323-2.813) | 0.01 |
|  | Rarely | 13 | 33 | 46 | 2.30 (1.38-3.84) | 0.01 |
| Washing hands before feeding | Always | 72 | 502 | 574 |  |  |
|  | Sometimes | 31 | 108 | 139 | 1.78 (1.22-2.60) | 0.03 |
|  | Rarely | 12 | 31 | 43 | 2.22 (1.31-3.77) | 0.03 |
| Use of soap for hand washing | Yes | 49 | 436 | 485 | I |  |
|  | No | 66 | 205 | 271 | 2.41 (1.72-3.38) | <0.001 |

CI: Confidence intervals, CPR: Crude prevalence ratio
