# Supplementary material for: Cryptosporidium infection and associated factors among diarrheic children under five years of age in Eastern Ethiopia
Source: PLoS Negl Trop Dis. 2025 Aug 5;19(8):e0013386. doi: 10.1371/journal.pntd.0013386 (PMC12349710; doi:10.1371/journal.pntd.0013386)
Supplement: S2 Table — (DOCX) [file pntd.0013386.s002.docx]

S2 Table. Studies reporting prevalence of *Cryptosporidium* infection among diarrheic children under five years of age in Sub-Saharan Africa countries

| Country | Number of children sampled | No (%) positive cases | Detection method | Authors. Year (reference) |
| --- | --- | --- | --- | --- |
| Angola | 344 | 101 (30.0) | Rapid test | Gasparinho et al. 2016 (1) |
| Botswana | 200 | 41 (20.5) | MZN | Kurenzvi et al., 2020 (2) |
| Ethiopia | 344 | 44 (12.8) | MZN | Abera et al., 2020 (3) |
| Ethiopia (substudy) | 58 | 3 (5.2) | IFAT | Kifleyohannes et al., 2022(4) |
| Ethiopia | 878 | 82 (9.0) | LED-AP | Johansen et al., 2022 (5) |
| Gabon | 241 | 31 (12.9) | PCR | Manouana et al., 2021 (6) |
| Gabon | 214 | 44 (21.0) | Rapid test | Krumkamp et al., 2021 (7) |
| Ghana | 410 | 47 (11.0) | Rapid test | Krumkamp et al., 2021(7) |
| Guinea-Bissau | 228 | 42 (18.4) | PCR | Mero et al., 2021 (8) |
| Kenya | 421 | 99 (23.5) | PCR | Mutai et al., 2020 (9) |
| Nigeria | 400 | 16 (4.0) | MZN | Agba et al., 2018 (10) |
| Kenya | 1778 | 195 (11.0) | ELISA | Delahoy et al., 2018 (11) |
| Kenya | 429 | 36 (8.4) | MZN | Wasike et al., 2015 (12) |
| Kenya | 243 | 77 (32.0) | ELISA | Deichsel et al., 2020 (13) |
| Madagascar | 209 | 25 (12.0) | Rapid test | Krumkamp et al., 2021 (7) |
| Malawi | 972 | 88 (9.1) | Rapid test | Bitilinyu-Bangoh et al., 2024 (14) |
| Mozambique | 319 | 35 (11.0) | MZN | Cassocera et al., 2022 (15) |
| Mozambique | 276 | 38 (13.9) | ELISA | Bauhofer et al., 2021 (16) |
| Mozambique | 831 | 28 (3.4) | MZN | Ferreira et al., 2020 (17) |
| Nigeria | 1185 | 274 (23.1) | ELISA | Balarabe-Musa & Dabo, 2022 (18) |
| Nigeria | 368 | 84 (22.8) | ELISA | Akpakpan et al., 2016 (19) |
| South Africa | 275 | 17 (6.0) | PCR | Potgieter et al., 2023 (20) |
| Sudan | 70 | 19 (27.1) | MZN | Tamomh et al., 2021 (21) |
| Tanzania | 462 | 68 (15.0) | Rapid test | Krumkamp et al., 2021 (7) |
| Tanzania | 723 | 113 (15.6) | PCR | Moyo et al., 2017 (22) |

Rapid test: lateral flow assay antigen detection, MZN: Modified Ziehl-Neelsen staining, IFAT: Immunofluorescent antibody test, LED-AP: Light-emitting diode-auramine-phenol fluorescence microscopy PCR: Polymerase chain reaction, ELISA: Enzyme-linked immunosorbent assay antigen detection

# References

1. Gasparinho C, Mirante MC, Centeno-Lima S, Istrate C, Mayer AC, Tavira L, et al. Etiology of diarrhea in children younger than 5 years attending the Bengo General Hospital in Angola. Pediatr Infect Dis J. 2016;35(2):e28-e34.

2. Kurenzvi L, Sebunya TK, Coetzee T, Paganotti GM, Teye MV. Prevalence of Cryptosporidium parvum, Giardia intestinalis and molecular characterization of group A rotavirus associated with diarrhea in children below five years old in Gaborone, Botswana. Pan Afr Med J. 2020;37:159.

3. Abera B, Hailu T, Beza L, Mulu W, Yizengaw E, Kibret M. Aetiology of acute diarrhoea and antimicrobial usage among children aged under five years at health centres in Bahir Dar, Ethiopia. Trop Doct. 2020;50(3):190-4.

4. Kifleyohannes T, Nødtvedt A, Debenham JJ, Tysnes KR, Terefe G, Robertson LJ. Cryptosporidium and Giardia infections in humans in Tigray, Northern Ethiopia: an unexpectedly low occurrence of anthropozoonotic transmission. Acta Trop. 2022;231:106450.

5. Johansen Ø H, Abdissa A, Bjørang O, Zangenberg M, Sharew B, Alemu Y, et al. Oocyst Shedding Dynamics in Children with Cryptosporidiosis: a Prospective Clinical Case Series in Ethiopia. Microbiol Spectr. 2022;10(4):e0274121.

6. Manouana GP, Byrne N, Mbong Ngwese M, Nguema Moure A, Hofmann P, Bingoulou Matsougou G, et al. Prevalence of Pathogens in Young Children Presenting to Hospital with Diarrhea from Lambaréné, Gabon. Am J Trop Med Hyg. 2021;105(1):254-60.

7. Krumkamp R, Aldrich C, Maiga-Ascofare O, Mbwana J, Rakotozandrindrainy N, Borrmann S, et al. Transmission of Cryptosporidium Species Among Human and Animal Local Contact Networks in Sub-Saharan Africa: A Multicountry Study. Clinical infectious diseases : an official publication of the Infectious Diseases Society of America. 2021;72(8):1358-66.

8. Mero S, Timonen S, Lääveri T, Løfberg S, Kirveskari J, Ursing J, et al. Prevalence of diarrhoeal pathogens among children under five years of age with and without diarrhoea in Guinea-Bissau. PLoS Negl Trop Dis. 2021;15(9):e0009709.

9. Mutai DC, Owili PO, Muga MA. Trend of cryptosporidium infection among children below 24 months in an informal urban settlement, Kenya. Open J Med Microbiol. 2020;10(3):153-61.

10. Agba A, Aken’Ova T, Audu P. Cryptosporidium infection among children attending some hospitals in Funtua Local Government área, Katsina state, Nigeria. Zoologist (The). 2018;16:1-5.

11. Delahoy MJ, Omore R, Ayers TL, Schilling KA, Blackstock AJ, Ochieng JB, et al. Clinical, environmental, and behavioral characteristics associated with Cryptosporidium infection among children with moderate-to-severe diarrhea in rural western Kenya, 2008–2012: The Global Enteric Multicenter Study (GEMS). PLoS Negl Trop Dis 2018;12(7):e0006640.

12. Wasike W, Kutima H, Muya M, Wamachi A. Epidemiology of Cryptosporidium spp. and other enteric parasites in children up to five years of age in Bungoma County, Kenya. J Biol Food Sci Res. 2015;4(1):1-6.

13. Deichsel EL, Hillesland HK, Gilchrist CA, Naulikha JM, McGrath CJ, Van Voorhis WC, et al., editors. Prevalence and correlates of cryptosporidium infections in kenyan children with diarrhea and their primary caregivers. Open Forum Infect Dis 2020: Oxford University Press US.

14. Bitilinyu-Bangoh JEV, Riesebosch S, Rebel M, Chiwaya P, Verschoor SP, Voskuijl WP, et al. Prevalence of Cryptosporidium and Giardia infections in under-five children with diarrhoea in Blantyre, Malawi. BMC Infect Dis. 2024;24(1):68.

15. Cassocera M, Bambo M, Anapakala E, Chissaque A, Sambo J, Langa JS, et al. High Frequency of Cryptosporidium hominis Infecting Infants Points to A Potential Anthroponotic Transmission in Maputo, Mozambique. PloS pathog. 2022:1-12.

16. Bauhofer AFL, Cossa-Moiane ILC, Marques SDA, Guimarães E, Munlela BA, Anapakala EM, et al. Intestinal protozoa in hospitalized under-five children with diarrhoea in Nampula - a cross-sectional analysis in a low-income setting in northern Mozambique. BMC Infect Dis. 2021;21(1):201.

17. Ferreira FS, Pereira F, Martins M. Intestinal parasitic infections in children under five in the Central Hospital of Nampula, Northern Mozambique. J Infect Dev Ctries. 2020;14(5):532-9.

18. Balarabe-Musa B, Dabo N. Epidemiology of Cryptosporidium and Rotavirus Diarrhoea in Children under the Age of Five in Asokoro District Hospital, Abuja, Nigeria. In: Francisco Cruz Sosa, editor. Innovations in Microbiology and Biotechnology. 72022. p. 104-25.

19. Akpakpan EE, Inabo HI, Aminu–Mukhtar M. Cryptosporidium parvum infection and nutritional status of children under-five years in some selected hospitals in Kaduna State, Nigeria. Int J Sci Res Publ 2016;6(1).

20. Potgieter N, Heine L, Ngandu JPK, Ledwaba SE, Zitha T, Mudau LS, et al. High Burden of Co-Infection with Multiple Enteric Pathogens in Children Suffering with Diarrhoea from Rural and Peri-Urban Communities in South Africa. Pathogens. 2023;12(2).

21. Tamomh AG, Agena AM, Elamin E, Suliman MA, Elmadani M, Omara AB, et al. Prevalence of cryptosporidiosis among children with diarrhoea under five years admitted to Kosti teaching hospital, Kosti City, Sudan. BMC Infect Dis. 2021;21(1):349.

22. Moyo SJ, Kommedal Ø, Blomberg B, Hanevik K, Tellevik MG, Maselle SY, et al. Comprehensive Analysis of Prevalence, Epidemiologic Characteristics, and Clinical Characteristics of Monoinfection and Coinfection in Diarrheal Diseases in Children in Tanzania. Am J Epidemiol. 2017;186(9):1074-83.
